# Supplementary material for: Cytokines Measured in Nasal Lavage Compared to Induced Sputum in Patients with Mild Cystic Fibrosis
Source: Int J Mol Sci. 2024 Oct 15;25(20):11081. doi: 10.3390/ijms252011081 (PMC11507901; doi:10.3390/ijms252011081)
Supplement: Supplementary file 1 [file ijms-25-11081-s001.zip › ijms-3262407-supplementary.pdf]

Table S1:
Comparison of inflammatory markers measured in serum, sputum (SP) and nasal lavage (NL) between the study groups. Results are expressed as median (range) with statistical analysis by Mann-Whitney U test.

Variable	Mild n=13	Severe n=19	p-value
IgG mg/dl	912 (415-1260)	1170 (464-1960)	**0.011**
IgG1 mg/dl	573 (327-830)	719 (351-1330)	**0.049**
IgG2 mg/dl	285 (56.4-335)	374 (75.7- 730)	**0.020**
IgG3 mg/dl	29.2 (15.4-55)	26.1 (10.1-59.5)	0.623
IgG4 mg/dl	76 (11.6-295)	97.6 (22.3-930)	0.287
IL-6 pg/ml	2.1 (1-6.6)	2.5 (0.6-384.4)	0.404
IL-8 pg/ml	19 (14-24)	15.0 (4-3650)	**0.049**
SP NE ng/ml	0.5 (0.2-1.7)	0.4 (0.2-2.2)	0.544
SP IL-1β pg/ml	17.2 (11-58.3)	39.5 (18-121)	**0.003**
SP IL-2 pg/ml	16.2 (10.5-98.8)	27.5 (14-92.5)	**0.009**
SP IL-6 pg/ml	29.8 (8-596)	51.2 (14-1121.5)	0.185
SP IL-8 pg/ml	2429.5 (23-10204.3)	4822.4 (65.8-18711)	0.232
SP IL-10 pg/ml	11.5 (10-16.5)	17.5 (11.3-34)	**<0.001**
SP IL-17a pg/ml	12.5 (9.5-24)	26.7 (12.5-49.5)	**<0.001**
NL NE ng/ml	1.1 (0.4-2.5)	0.6 (0.2-3.0)	**0.016**
NL IL-1β pg/ml	22.5 (12.5-73)	21 (10-66.3)	0.372
NL IL-2 pg/ml	24 (15-29)	21.5 (15-27.5)	0.525
NL IL-6 pg/ml	54.4 (19.5-365.8)	24.5 (16.3-99)	**0.018**
NL IL-8 pg/ml	1796.8 (433.8-7735)	760.2 (86.8-3479.5)	**0.043**
NL IL-10 pg/ml	10 (8.3-11)	9 (6-12)	0.122
NL IL-17a pg/ml	14 (11-67.3)	14.5 (8-70)	0.420

Table S2:

Most striking correlations of inflammatory markers with each other and with clinical parameters.

Variable	2	3	4	5	6	7	8	9	10	11	12	13	14	15
**1** NL NE	-.32	-.02	0	-.09	.35	.04	.04	-.24	-.27	-.21	-.01	.18	.24	-.11
	**.64***	**.77****	.41	.16	**.78****	.26	-.09	.20	-.20	**.59***	-.08	.01	.16	-.08
**2** NL IL-1β		.02	-.12	**.49***	-.16	.18	.19	.03	-.03	.11	-.15	-.10	-.19	-.02
		**.66***	.04	.16	**.68***	**.85*****	-.32	.36	-.01	**.82****	-.15	.26	.05	-.27
**3** NL IL-6			**.87*****	.32	.16	.11	.11	-.25	.15	-.01	-.17	-.43	-.41	.13
			.19	-.13	**.64***	.11	.01	-.19	**-.60***	.40	.13	-.23	-.28	-.29
**4** NL IL-8				.12	.01	-.07	-.07	-.35	.18	-.12	.07	-.34	-.29	.15
				-.05	.13	-.24	-.41	-.13	.07	.01	-.02	-.37	-.19	-.31
**5** NL IL-10					-.21	.06	.07	-.23	-.43	-.03	-.45	**-.58***	**-.67****	-.44
					-.03	-.05	-.27	.30	.07	-.11	-.55	.27	.38	-.55
**6** TNFα						**.59****	**.59****	.03	.21	.40	-.07	.04	.15	.17
						.48	-.28	.30	-.03	**.84*****	-.20	-.31	-.30	-.21
**7** IL-6							**1*****	-.14	.05	.23	.05	.27	.22	.24
							-.27	.08	-.07	**.70****	-.09	.43	.11	.01
**8** IL-8								-.15	.04	.22	.05	.27	.22	.24
								.01	-.15	-.11	.14	.16	.23	.13
**9** IgG1									.38	-.11	-.20	.02	.20	**.50***
									**.75****	.34	-.20	-.15	.14	-.10
**10** IgG2										.27	.15	.12	.22	**.64****
										.09	-.08	-.47	-.19	.13
**11** IL-10											.01	-.04	-.01	-.08
											-.18	-.12	-.28	-.21
**12** SP IL-2												**.95*****	**.97*****	.28
												**.76****	**.83*****	.48
**13** SP IL-10													**.96*****	.24
													**.83*****	.48
**14** SP IL-17a											-			.36
														.44
**15** SP IL-1β														-

Note: Spearman correlation coefficient is given on top for severe patients, on bottom for mild patients. Significant values are shown in bold (red for severe, blue for mild). *p<0.05, **p<0.01, ***p<0.001.
